# Biomarker development for translational geroscience: Considerations for a strategic framework focusing on early clinical development

**DOI:** 10.1111/acel.13837

**Published:** 2023-04-06

**Authors:** Jason L. Sanders

**Affiliations:** ^1^ Vertex Pharmaceuticals Inc. Boston Massachusetts USA

The expectation that geroscience will allow humans to age optimally continues to generate excitement among a growing community of professionals and non‐professionals, including high profile personalities in business. But the stakes are high, and if expectations grow from fanciful ambition rather than data, failure will lead to bewilderment, disillusionment, and dampened public support. The core mission of geroscience is to improve healthspan by targeting mechanisms associated with biological aging; whether this is possible in humans remains at present a hypothesis.

Geroscience clinical trials will be the experiments that provide the strongest data to pressure test the geroscience hypothesis. While these trials can be described simply, adding to their public appeal, those of us involved in the conception of trial design increasingly recognize that unless geroscience clinical trials are conducted with rigor like traditional disease‐based clinical trials, the scientific community, and, perhaps more importantly, regulatory agencies, will lose their enthusiasm and support for geroscience‐based intervention that might have merited development. In this editorial, I briefly describe some methodological complexities in geroscience clinical trial design and argue for a strategic approach to de‐risk decisions which must be made during early clinical development.

Among individuals who are, say, 55–80 years old, who may be most likely to enroll in a geroscience clinical trial, hard clinical outcomes such as death and incident cardiovascular disease, cancer, and dementia, tend to occur at low rates. Subsequently, geroscience clinical trials will require, on average, longer treatment periods than typical disease‐based clinical trials to accumulate enough hard events to achieve adequate statistical power. The theorized pleiotropic effect of geroscience interventions (i.e., common mechanisms drive multiple morbid outcomes) could mean that effect sizes on any specific outcome may be modest, making it difficult to predict which age‐associated outcomes will benefit from treatment. This contrasts with a traditional disease‐based trial, where outcomes typically occur at higher rates and interventions often hinge on narrowly defined targets, making trials more efficient.

Trialists may find it appealing to develop *composite endpoints*, which are defined by regulatory agencies as the occurrence or realization in a patient of any one of a set of specified components and measured as the time to developing the first event of any one occurrence within the composite endpoint (FDA, 2022). Composite endpoints lend themselves well to interventions aimed at preventing or delaying morbidity, a key goal of geroscience, and often have face validity when components are hard clinical outcomes, such as incident cardiovascular disease, cancer, dementia, or death. Composite endpoints are useful when each of the component endpoints occurs at a low rate; by increasing the overall event rate of the composite endpoint, trial size and duration may be reduced. Statistical adjustment for multiplicity (as is necessary with co‐primary endpoints) is unwarranted, and therefore, a single statistical test can be performed on the composite endpoint. Seemingly, composite endpoints provide the rigor that geroscience trials need while preserving efficiency.

However, composite endpoints can be tricky. We must decide which components to include in the composite, a particular problem for geroscience where there is little agreement on the most significant endpoints (e.g., molecular biomarkers of accelerated aging, worse tissue pathology, incident diagnosed disease, functional decline, and death). By default, each component is given equal statistical weight, though each component may differ in clinical impact. Furthermore, a significant therapeutic effect on the overall composite will mask the effect on each component—if this determination is necessary, it should be pre‐specified with adjustments for multiplicity, which can impact trial efficiency. More troubling, a positive treatment effect could be found with the overall composite endpoint despite some component(s) potentially being adversely affected, obscuring a potential harm signal. Because geroscience‐based interventions are intended to have pleiotropic effects, there may be multiple adverse events of special interest that require detection across organ systems.

An alternative to a composite endpoint is a *multicomponent endpoint*, which is a within‐subject combination of two or more components, tabulated according to pre‐specified rules, such as a summary scale (e.g., frailty index, fatiguability, and physiologic index of comorbidity) (FDA, 2022; Glynn et al., 2015; Newman et al., 2008). A treatment effect depends on altering anywhere from one to all components. If all components trend in the same direction within a patient in response to treatment, it can suggest a positive effect. Unfortunately, low concordance between changes in each component can negate gains in power, and the same issues remain regarding selecting each component and accounting for multiplicity if there is interest in each component. Developing novel multicomponent endpoints often involves determining a minimal clinically important difference (MCID); for a geroscience‐based multicomponent endpoint, the validation process itself could take many years if each component changes slowly with aging.

With these pitfalls, how should we proceed with geroscience trial design? Although no trial design exists without risk, we have a say in how much risk we can tolerate before beginning a trial. Enormous resources and time can be spent attempting to generate data to de‐risk decisions on trial design. But the alternative is worse: beginning trials at high risk, based on scant data, excitement, and ambition, could result in early trial failures that negatively affect the field. Perhaps a balance can be achieved by guiding data generation strategically with biomarkers.

Strategic development of biomarkers represents an opportunity to accelerate geroscience clinical trials, particularly in early phases to satisfy the requirements for rigor and efficiency. Highly dynamic and predictive intermediate endpoints captured by validated biomarkers could provide confidence in earlier trial phases to strengthen commitment to late‐stage registrational trials. In this scenario, biomarkers are not meant to be regulator‐approved surrogate endpoints with which to accelerate registration. Rather, they are meant to accelerate internal decision‐making (i.e., go/no‐go) through proof‐of‐concept studies. For example, efficiencies could be gained with respect to determining who might best respond to a geroscience intervention (i.e., *Patient selection*); what treatment dose associates with a pre‐defined magnitude of change in on‐ and off‐target biomarkers (i.e., *Pharmacodynamic response*); what magnitude of change in biomarkers associates with a clinically significant response (i.e., *Clinical efficacy*); and what early safety signals might exist (i.e., *Safety and tolerability*).

Pharmaceutical industry approaches may provide guidance here through strategic biomarker development, with each proposed biomarker explicitly evaluated with respect to its intended use in early clinical development. This differs from the common practice of, for example, conducting *in vitro* and epidemiological analyses to observe biomarker patterns and using those patterns as rationale that a biomarker has value in a clinical trial. Sticking to a strategic framework forces us *a priori* to study a biomarker for patient selection, pharmacodynamic response, clinical efficacy, or safety and tolerability. As I will explain shortly, if we intend to rely on biomarker readouts to de‐risk go/no‐go decisions regarding clinical trial design and interpreting trial results, the most actionable information will be generated if we plan experiments around each biomarker's intended use (detailed in Table 1).

**TABLE 1:** Considerations for each strategic step of biomarker development.

Biomarker use	Considerations for use
Patient selection	Does therapeutic effect depend on patient characteristics? Is genetic, molecular, or other stratification part of standard of care? Is a companion diagnostic needed? What is the minimum scientific, analytical, and clinical data package required for a companion diagnostic? What is the development strategy to deliver this data package for the companion diagnostic?
Pharmacodynamic response	What is the drug's mechanism of action? How does mechanism of action impact measuring the association between therapeutic target and phenotypic effect? In what matrix and with what methods should the target and phenotype be measured? Are measurement methods commercially available, or do they require development?
Clinical efficacy	What are the most important endpoints for mid/late‐stage trials? How can preclinical data and first‐in‐human results be used to predict effects in mid/late‐stage trials? How accurate and precise are predictions of effects in mid/late‐stage trials using preclinical and first‐in‐human data? What extent of validation is required for endpoints used in internal vs. external decision‐making?
Safety & tolerability	Based on mechanism of action, patient population for intended use, and disease characteristics, what are the most likely and concerning safety signals that must be measured?

If we evaluate biomarkers within a strategic framework, it leads us to ask different questions that guide experimental design. For example, using a framework to develop biomarkers for a trial of senolytics would force us to ask: “*Do participants with different levels of DNA methylation, mitochondrial respiration, and SASP respond differently to exposure/treatment with senolytics?* (Patient selection) *What biomarker cut points should be used to exclude those unlikely to benefit from senolytics?* (Patient selection) *What amount of senolytics is associated with a pre‐defined magnitude of change in these biomarkers?* (Pharmacodynamic response) *What magnitude of change in the pre‐selected biomarkers associates with a clinical response in humans that might be observable during the length of time planned for a clinical trial's treatment period?* (Clinical efficacy) *Have changes in these biomarkers, to this amount, in the proposed population for enrollment, been associated with specific harm?* (Safety and tolerability)” At the conclusion of the strategic biomarker development program, I argue that data would be more interpretable and thus actionable to support (or not) use during early clinical development.

Currently, there are no widely accepted, specific, sensitive, accurate, reproducible, and clinically validated biomarkers of aging in humans that meet regulatory standards—they are all exploratory (Justice et al., 2018). We are also all aware that some within our field, including notable academics, have lent their credentials to support a new generation of unregulated products and services sold directly to consumers that purport to offer clear, actionable information about one's health and behaviors, in many ways forgetting the disillusionment that communities experienced with similar approaches in nutraceuticals and the nutrition industry. These products are frequently costly biomarker tests backed by a thin veneer of scientific certainty. Furthermore, these measurements are often coupled to “recommended” (and sometimes costly) interventions, such as supplements, off‐label use of existing medications, and trials of unproven technologies, to allay a consumer's fears produced by a “conclusive” biomarker test. Surely, as a scientific community, we can agree that endorsement of these products and services does a disservice to the geroscience mission of advancing healthspan. If we do not hold ourselves and each other to the highest standards, we (geroscientists) run the risk of being viewed as peddlers of snake oil (which can erode public confidence in geroscience), and potentially cause medical harm directly to patients or indirectly through opportunity lost from evidence‐based trials.

In conclusion, strategically developed and validated biomarkers may help us overcome some of the biggest roadblocks in geroscience clinical development. I urge our community of geroscientists to optimize the probability of successfully validating and applying translational biomarkers by using a strategic biomarker development framework. Let us collectively develop best practices and guidelines to give the world its best shot at realizing the promise of geroscience‐based therapies: increased healthspan.

## CONFLICT OF INTEREST STATEMENT

JLS is an employee of and holds stock in Vertex Pharmaceuticals Inc. The views expressed within are solely those of the author and not those of Vertex or Aging Cell.
